# Risk of bacterial contamination of bone harvesting devices used for autogenous bone graft in implant surgery

**DOI:** 10.1186/1746-160X-9-3

**Published:** 2013-01-11

**Authors:** Megumi Takamoto, Masaaki Takechi, Kouji Ohta, Yoshiaki Ninomiya, Shigehiro Ono, Hideo Shigeishi, Misato Tada, Nobuyuki Kamata

**Affiliations:** 1 Department of Oral & Maxillofacial Surgery, Graduate School of Biomedical & Health Sciences, Hiroshima University, 1-2-3 Kasumi, Minami-Ku, 734-8553, Hiroshima, Japan

**Keywords:** Bacterial contamination, Intraoral autogenous bone graft, Device for collecting bone debris

## Abstract

**Background:**

Various instruments have been developed for collecting bone debris during intraoral autogenous bone graft procedures in implant surgery. The aim of this study was to quantitatively determine the degree of contamination in bone debris collected by different devices.

**Methods:**

Twelve patients underwent autogenous bone collection using a bone chisel, bone scraper, trephine drill, and bone filter during bone augmentation surgery as a part of implant therapy, and the total bacterial count in bone debris collected by each was determined.

**Results:**

Following anaerobic incubation, bacterial colony formation was found in all of the samples. The mean colony forming units (CFU)/g in samples collected by the trephine drill was found to be significantly lower than that of samples obtained with the other devices, while those values for samples collected by the bone scraper and bone filter was significantly higher as compared to the bone chisel and trephine drill.

**Conclusion:**

The bacterial levels may still carry the infection risk. Thus prophylactic antibiotic therapy maybe indicated when using bone particles for intraoral augmentation procedures.

## Introduction

Autogenous bone graft is the gold standard method for treating bone defects and reconstructing alveolar bone as a part procedure of implant therapy, because it does not produce immunologic rejection and it contains osteoinductive components [1,2]. Small bones used for such grafting are commonly obtained intra-oral sources, such as the mandibular ramus and retromolar area. It has been reported that use of intraoral donor sites has several advantages as compared to extraoral sites, including reduced operation and hospitalization time, and no cutaneous scarring [3,4].

Several devices have been developed for collecting intraoral autogeneous bone graft to date. Bone filter, one of these devices, in which filters are placed in the surgical suction system, collects the bone debris produced during bone drilling for implant site preparation [5]. However, the possible risk of iatrogenic contamination has been reported in bone samples collected by this system, which may lead to infection or failures of implant therapy [6,7]. Although other devices also may carry a risk of contamination, there are few reports of bacterial contamination in bone particles collected by various devices.

In the present study, we focused on the bacterial contamination of bone debris collected by several devices for intraoral autogeneous bone graft. To quantitatively determine the degree of contamination in the bone samples, we determined the total bacterial count in bone debris collected by a bone chisel, bone scraper, bone filter, and trephine drill during intraoral autogenous bone graft procedures for implant surgery.

## Materials & methods

### Devices

**Figure 1:**
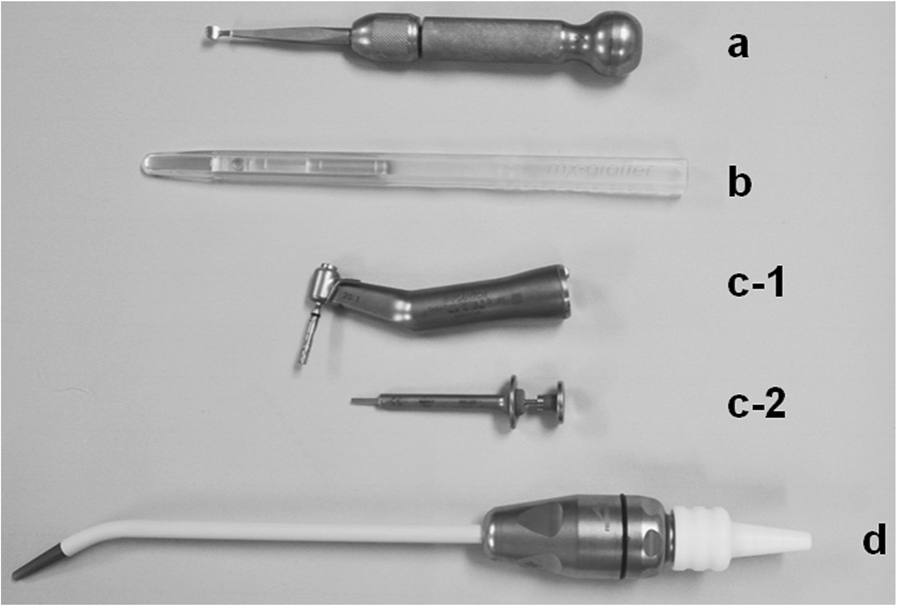
**Devices to collect bone debris for autogenous bone graft procedures. (a)** Bone chisel **(b)** Bone scraper **(c-1)** Trephine drill and contra-angle hand-piece **(c-2)** Syringe used for injection. **(d)** Bone filter.

We used the following devices in the present study. A bone chisel, (EZ Bone Shaver; Omic Corporation, Shiga, JAPAN), a handheld chisel-type of instrument equipped with a spoon-shaped end, which makes possible to incise and widen ridges, and scrape bone tip from cortical bone surfaces (Figure 1a).

A bone scraper, (MX-Grafter; Maxilon Laboratories, NH, USA), which consists of a blade body, and collection chamber. The hardened hollow ground blade makes point contact with relatively flat bone surfaces when it is held at an angle of approximately 5° to 50° to the surface. In use, the bone scraper is pulled along the cortical bone surface to shave the bone, and bone tips advance into the storage chamber for collection and strage (Figure 1b).

**Figure 2:**
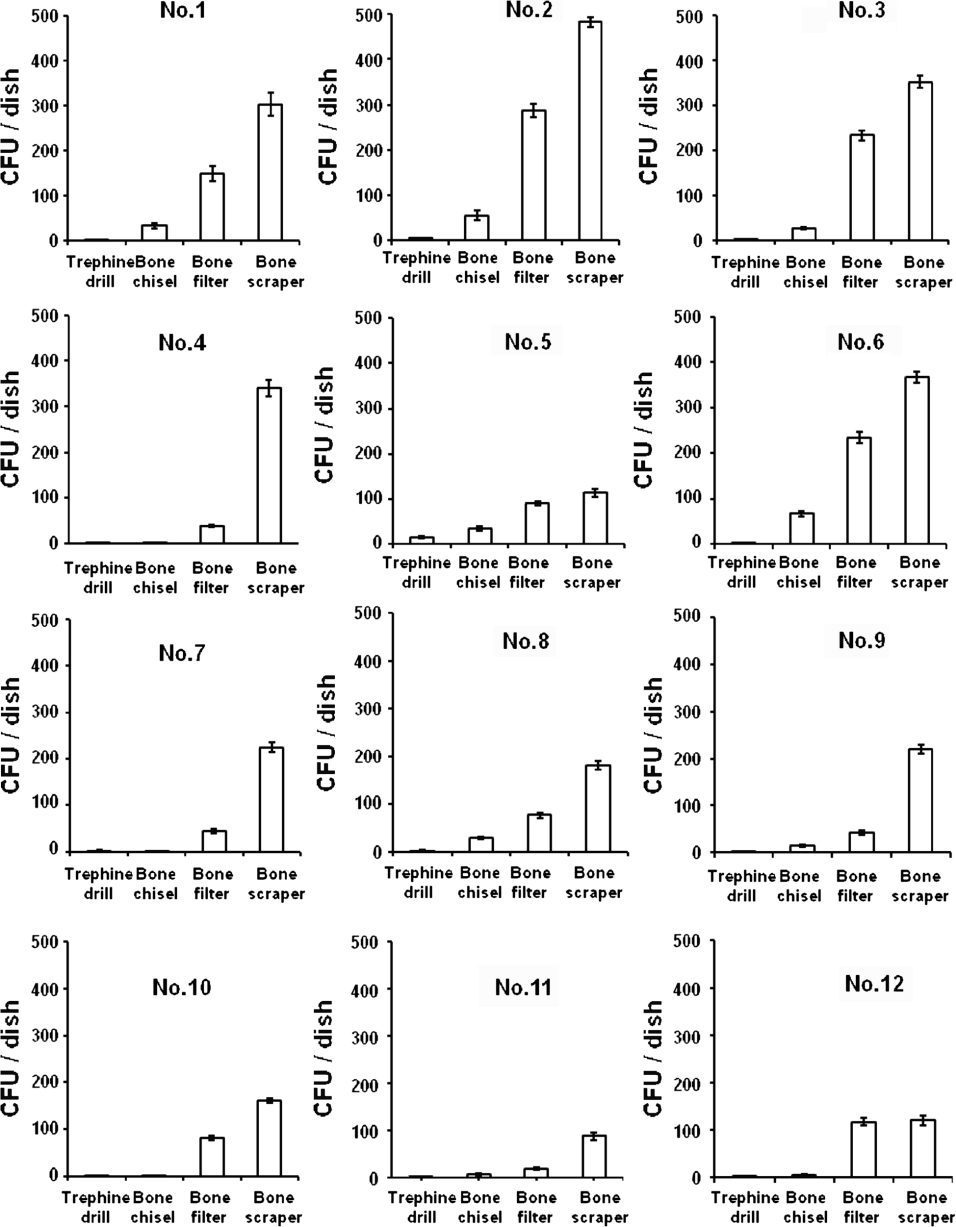
**Number of CFU/sample of autogenous bone collected by the devices from 12 patients.** The total CFU per dish for each device were determined.

A trephine drill, (K-trephine System; Dentak Corporation, France), which is comprised of a tubular body fitted to a contra-angle hand-piece (Figure 1c-1). The drill is used to make holes in cortical bone at a low speed turning, and crushed bony gradually fills up the trephine tube. For later application to the graft site, the blade drill is removed from the trephine tube and then the tube is fitted onto a syringe adapted for injection (Figure 1c-2).

A bone filter, (Frios Bone-collector; Friadent GmbH, Mannheim, Germany), a filter system placed in a suction device to collect bone debris produced during drilling for implant site preparation (Figure 1d). Bone debris is collected in the filter using a stringent aspiration protocol, which restricts the suction tip to only collect bone, blood, and irrigant, such as sterile saline from the surgical site [7]. Bone samples are removed from the bone trap using a sterile curette (Figure 1d).

### Subjects

The study population was 12 patients (mean 50.1 years old, range 20–67 years) undergoing intraoral autogenous block bone graft surgery (onlay or veneer graft) for implant treatment at Hiroshima University Hospital. The study protocol was reviewed and approved by the Ethical Committee of Hiroshima University Hospital. All patients were informed regarding the risks of the surgical procedure and each gave voluntary written consent prior to taking part in the study.

### Surgical course

All surgeries were performed by the same surgeon in an operation room. Both the surgeon and assistants wore sterile gowns and gloves, while the patients were fully covered with sterile drapes in the usual manner, and iodine was used for mouth rinse. The lips and perioral facial skin areas of the patients were disinfected with benzalkonium chloride. In addition, antibiotics were administered for surgical wound infection during and after the surgery.

An incision was made medial to the external oblique ridge in an anterior direction and terminated in the first molar area. Following soft tissue flap elevation, a block of bone was harvested for use as onlay or veneer graft from the anterior border of the mandibular ramus using a small round bar and fissure bur. A bone filter was simultaneously used to collect bone debris produced during the drilling for block bone harvesting, while debris was also collected from similar areas using the other devices in a randomed order, and used for autogenous bone graft. From each leftover sample after bone graft, 50 mg was obtained and placed into 5 ml of PBS, then stored in a refrigerator and microbiologically examined within 4 hours of the procedure.

### Laboratory course

As previously reported by Lambrecht *et al.*[8], the samples were mixed on a vortex for 1 minute, then diluted 10-fold with PBS. Next, 100 μl of each suspension sample was directly applied directly onto BHI agar plates (Becton Dickinson Co., Cockeysville, MD, USA). The plates (n=3) were incubated at 37°C for 2 days under an anaerobic condition using an Anaero Pouch System (Mitsubishi Gas Chemical,Tokyo, Japan). Bacterial colonies were counted on each dish, and colony forming units (CFU) per gram were calculated from the total number of CFU per dish for the samples from each patient. Bacterial species was identified from anaerobic culture of bone debris by SRL, Inc (Tokyo, Japan) using a Rapid Ana II System (Amco, Tokyo, JAPAN).

### Statistical analysis

Data were statistically evaluated using one-way ANOVA followed by Dunnett’s multiple comparison test and the results are presented as the mean ± standard deviation. The difference between means was considered significant at *P<*0.05.

## Results

**Figure 3:**
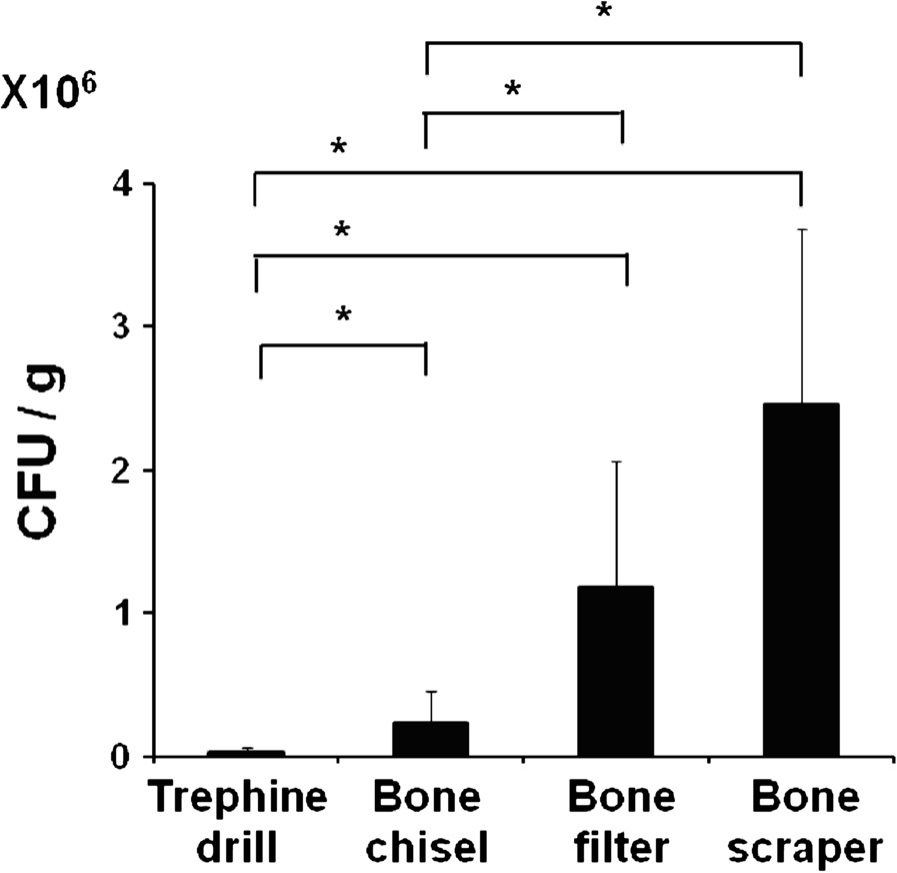
**Mean CFU/g of autogenous bone collected by the 4 devices.** The mean CFU per gram in each group were calculated based on the total CFU per dish. *Significantly different from other differential devices (Dunnett’s test, p < 0.05).

None of the patients had symptoms of infection or implant failure after autogenous bone graft. Following anaerobic incubation, bacterial colonies were found on all agar plates applied with bone samples from the patients. Although the values for CFU/dish varied, there was a clear correlation between those values and the type of device used to collect the sample. The values for samples obtained by trephine drill were always the lowest, followed in order by those obtained by the bone chisel, bone collector, and bone scraper for all of the patients (Figure 2). The mean CFU/g of the samples collected by the trephine drill was significantly lower than all others, while those of samples collected by the bone scraper and bone filter were significantly higher than those of samples collected by the bone chisel and trephine drill (Figure 3). Bacterial species were identified in bone debris collected by each devises from 6 patients. Streptococcus spp and Neisseria spp were found in all of the samples (Table 1). However, no specific bacterium showed a prevalent occurrence among the devices (Data not shown).

**Table 1:** Distribution of bacterial species in the debris collected by the devices

**Species**	**Sample numbers**
Streptococcus spp	1, 2, 3, 4, 5, 6
Neisseria spp	1, 2, 3, 4, 5, 6
Actinomyces spp	1, 2, 3
Capnocytophaga spp	3,5
Prevotella spp	4
Staphylococcus spp	3
Hemophilus spp	5

## Discussion

Devices for collecting small bone debris from intraoral sites are useful in situations when small amounts of bone are needed for augmentation procedures in implant treatment. A bone filter is widely used as a suction device for collecting bone debris produced during bone drilling for implant site preparation [9,10]. However, bacterial contamination from the host oral environment has been reported in samples collected by such a filter. Young *et al.* reported bacterial contamination of 1.5X10^8^ CFU/g from bone collected by a bone filter, even when a stringent aspiration method was utilized [6]. In the present study, a mean 2.4 X10^6^ CFU/g of bacterial contamination was found in bone debris collected by such a bone filter. These high bacterial colony levels may have been caused by the inadequate aspiration of salivary-tissue fluid in spite of strict aspiration.

A bone scraper has been reported to be an adequate harvesting device for limited intraoral augmentation [11]. In that study, the device was used for autogenous bone graft for implant treatment in the maxilla, and no implant failures or signs of bone resorption were noted during the second stage surgery, as well as in follow-up examinations [11]. In the present study, the mean CFU/g of bone debris collected by the bone scraper was higher as compared to the bone chisel and trephine drill. Since a similar device was also reported as a useful instrument for intraoral bone graft [12], we compared the bacteria levels of bone debris collected by another bone scraper similar to the device used in this study, with nearly the same level found in bone debris collected by both devices (data not shown). Direct trapping of salivary fluid into the chamber by large movements of this instrument along the bone surface may be related to the high levels of bacterial contamination.

The mean CFU/g of samples collected by trephine drill was the lowest among 4 tested devices. A trephine bone drill moves perpendicular to cortical bone within the narrow space used to make a hole, thus there may be fewer opportunities for exposure to salivary-tissue fluid responsible for bacterial contamination.

The bacteria species isolated from bone debris in this study are commonly detected in salivary flora [13], and related to a variety of infections, including bone infections [8,14], with Streptococcus viridans species primarily seen in subacute bacterial endocarditis cases [15,16]. Thus, use of bone collected from the devices investigated in this study for intraoral graft may be contraindicated for treatment of immunosuppressed patients.

Use of chlorhexdine for irrigation instead of sterile saline is considered to reduce the risk of microbial contamination during intraoral augmentation procedures. When collecting bone by a bone filter, oral rinsing with chlorhexidine has been shown to reduce the quantity of oral microbial populations [6,15], though those findings do not strongly support the advantage of a preoperative chlorhexidine mouth rinse for reducing bacterial levels in bone collected by bone filter [6,15]. Furthermore, another study noted that the effects of chlorhexidine on osteogenic potential of bone collected by a bone filter and wound healing are controversial [17]. Grafting procedures by using autogenous bone collected by various devises have to continue to undergo refinement with the aim of further reducing initial bacterial contamination levels.

In the present study, we found that the levels of bacterial contamination of bone varied among 4 devices used for intraoral autogenous bone graft, though none of the patients had signs of infection after undergoing implant surgery. Although the trephine drill showed the lowest number of bacterial colonies in bones collected by the devices, it is invasive in some patients when an adequate depth is needed to cut into cortical bone [18]. Thus, the choice of bone harvesting devices should be considered based on their individual advantages and disadvantages.

Our results suggest that use of bone particles collected by the tested devices may carry the risk of infection during intraoral augmentation procedures. Therefore, prophylactic antibiotic therapy may be indicated, while development of a bone graft substitute without the requirement of autogenous bone graft, or improvements in implant surfaces to inhibit bacterial adhesion but not bone formation and /or osseointegration may be necessary in the future.

## Conclusion

We demonstrated that the levels of bacterial contamination of bone varied among 4 devices used for intraoral autogeneous bone grafting procedures. The bacterial levels may still carry infection risk and prophylactic antibiotic therapy may be indicated when using bone particles collected by the devices in this study for intraoral augumentation procedures.

## Competing interest

The authors declare that they have no competing interests.

## Authors’ contribution

TM, TM, OK conceived the study design and wrote manuscript. YN, SH, OS performed surgery and carried out bacterial analysis. MT and KM participated in the design of the study and helped to draft the manuscript. All authors read and approved the final manuscript.
